# National policies on the management of latent tuberculosis infection: review of 98 countries

**DOI:** 10.2471/BLT.17.199414

**Published:** 2018-02-05

**Authors:** Ann Jagger, Silke Reiter-karam, Yohhei Hamada, Haileyesus Getahun

**Affiliations:** a University of California Berkeley, School of Public Health, Berkeley, California, United States of America.; b Global Tuberculosis Programme, World Health Organization, Avenue Appia 20, 1211 Geneva 27, Switzerland.

## Abstract

**Objective:**

To review policies on management of latent tuberculosis infection in countries with low and high burdens of tuberculosis.

**Methods:**

We divided countries reporting data to the World Health Organization (WHO) Global Tuberculosis Programme into low and high tuberculosis burden, based on WHO criteria. We identified national policy documents on management of latent tuberculosis through online searches, government websites, WHO country offices and personal communication with programme managers. We made a descriptive analysis with a focus on policy gaps and deviations from WHO policy recommendations.

**Findings:**

We obtained documents from 68 of 113 low-burden countries and 30 of 35 countries with the highest burdens of tuberculosis or human immunodeficiency virus (HIV)-associated tuberculosis. Screening and treatment of latent tuberculosis infection in people living with HIV was recommended in guidelines of 29 (96.7%) high-burden and 54 (79.7%) low-burden countries. Screening for children aged < 5 years with household tuberculosis contact was the policy of 25 (83.3%) high- and 28 (41.2%) low-burden countries. In most high-burden countries the recommendation was symptom screening alone before treatment, whereas in all low-burden countries it was testing before treatment. Some low-burden countries’ policies did not comply with WHO recommendations: nine (13.2%) recommended tuberculosis preventive treatment for travellers to high-burden countries and 10 (14.7%) for patients undergoing abdominal surgery.

**Conclusion:**

Lack of solid evidence on certain aspects of management of latent tuberculosis infection results in national policies which vary considerably. This highlights a need to advance research and develop clear, implementable and evidence-based WHO policies.

## Résumé

**Objectif:**

Passer en revue les politiques relatives à la prise en charge de l'infection tuberculeuse latente de pays faiblement ou fortement touchés par la tuberculose.

**Méthodes:**

Nous avons séparé les pays qui transmettent des données au Programme mondial de lutte contre la tuberculose de l'Organisation mondiale de la Santé (OMS) selon qu'ils présentaient une charge de morbidité tuberculeuse faible ou élevée, suivant les critères de l'OMS. Nous avons trouvé les politiques nationales pour la prise en charge de la tuberculose latente grâce à des recherches en ligne, aux sites Internet des gouvernements, aux bureaux de l'OMS dans les pays et à des communications personnelles avec des responsables de programmes. Nous avons fait une analyse descriptive axée particulièrement sur les lacunes des politiques et leurs écarts des recommandations de l'OMS.

**Résultats:**

Nous avons obtenu des documents de 68 pays sur 113 ayant une faible charge de morbidité tuberculeuse et de 30 pays sur 35 ayant les plus fortes charges de morbidité dues à la tuberculose ou à la tuberculose associée au virus de l'immunodéficience humaine (VIH). Le dépistage et le traitement de l'infection tuberculeuse latente chez les personnes vivant avec le VIH étaient recommandés dans les directives de 29 pays (96,7%) à la charge de morbidité élevée et de 54 pays (79,7%) où cette charge était faible. Le dépistage des enfants de moins de 5 ans en contact familial avec la tuberculose était prévu dans 25 pays (83,3%) à la charge de morbidité élevée et dans 28 (41,2%) à la charge de morbidité faible. Dans la plupart des pays à la charge de morbidité élevée, seule la recherche des symptômes avant traitement était recommandée, tandis que tous les pays à la charge de morbidité faible recommandaient un dépistage avant traitement. Les politiques de certains pays à la charge de morbidité faible ne suivaient pas les recommandations de l'OMS: neuf (13,2%) recommandaient un traitement préventif contre la tuberculose pour les personnes se rendant dans des pays à la charge de morbidité élevée et 10 (14,7%) pour les patients subissant une chirurgie abdominale.

**Conclusion:**

En l'absence de preuves solides concernant certains aspects de la prise en charge de l'infection tuberculeuse latente, les politiques nationales varient considérablement les unes des autres. Cela met en lumière la nécessité de faire progresser la recherche et, pour l'OMS, d'élaborer des politiques claires, applicables et fondées sur des éléments probants.****

## Resumen

**Objetivo:**

Analizar las políticas sobre el tratamiento de la infección de tuberculosis latente en países con niveles bajos y elevados de tuberculosis.

**Métodos:**

Dividimos a los países que informan datos al Programa Global de Tuberculosis de la Organización Mundial de la Salud (OMS) en nivel de tuberculosis bajo y elevado, conforme a los criterios de la OMS. Identificamos los documentos de políticas nacionales sobre el tratamiento de la tuberculosis latente a través de búsquedas en línea, sitios web gubernamentales, sedes nacionales de la OMS y comunicación personal con los administradores del programa. Realizamos un análisis descriptivo enfocado en las desviaciones y los vacíos de las políticas con respecto a las recomendaciones de políticas de la OMS.

**Resultados:**

Obtuvimos documentos de 68 de 113 países con nivel bajo y de 30 de 35 países con los niveles más elevados de tuberculosis o tuberculosis asociada con el virus de la inmunodeficiencia humana (VIH). En las pautas de 29 (96,7%) países con nivel elevado y 54 (79,7%) países con nivel bajo se recomendaron exámenes de detección y tratamiento de la infección de tuberculosis latente en personas que viven con el VIH. La política de 25 (83,3%) países con nivel elevado y de 28 (41,2%) países con nivel bajo se basó en exámenes de detección en niños menores de cinco años de edad con contacto doméstico con tuberculosis. En casi todos los países con nivel elevado, la recomendación consistió únicamente en el examen de detección de los síntomas antes del tratamiento, mientras que, en todos los países con nivel bajo, esta consistió en pruebas antes del tratamiento. Las políticas de algunos países con nivel bajo no cumplen con las recomendaciones de la OMS: nueve (13,2%) recomendaron el tratamiento preventivo de la tuberculosis para viajeros que se dirijan a países con nivel elevado y 10 (14,7%) para pacientes que se sometan a una cirugía abdominal.

**Conclusión:**

La falta de pruebas sólidas sobre ciertos aspectos del tratamiento de la infección de tuberculosis latente da lugar a políticas nacionales que varían considerablemente. Esto subraya la necesidad de realizar investigaciones avanzadas y desarrollar políticas de la OMS que sean claras, implementables y empíricas.

## ملخص

**الغرض:**

مراجعة السياسات المتبعة للتعامل مع عدوى السل الكامنة في البلدان التي تعاني من معدلات منخفضة للإصابة بمرض السل والبلدان التي تعاني من معدلات مرتفعة للإصابة بالمرض.

**الطريقة:**

قمنا بتقسيم البلدان التي قدمت بيانات للبرنامج العالمي التابع لمنظمة الصحة العالمية والمعني بمكافحة مرض السل إلى بلدان لديها معدلات منخفضة وأخرى تعاني من معدلات مرتفعة للإصابة بالسل وذلك استنادًا لمعايير منظمة الصحة العالمية. وقد حددنا وثائق السياسات الوطنية للتعامل مع مرض السل الكامن من خلال البحث على شبكة الإنترنت والمواقع الإلكترونية للحكومات والمكاتب القُطرية لمنظمة الصحة العالمية والاتصال الشخصي مع مديري البرنامج. كما قمنا بإجراء تحليل وصفي يركز على الثغرات وحالات الحيود في السياسات المتبعة عن توصيات السياسات لمنظمة الصحة العالمية.

**النتائج:**

حصلنا على وثائق من 68 بلدًا من أصل 113 بلدًا لديها معدلات منخفضة للإصابة بمرض السل أو مرض السل المصاحب لفيروس عوز المناعة البشري (HIV)، كما حصلنا على وثائق من 30 بلدًا من أصل 35 بلدًا تعاني من معدلات مرتفعة. وقد وردت توصيات بفحص وعلاج عدوى السل الكامنة لدى المصابين بفيروس عوز المناعة البشري في المبادئ التوجيهية المتبعة لدى 29 بلدًا (بواقع 96.7‏%) تعاني من ارتفاع معدلات الإصابة بالمرض، وكذلك في 54 بلدًا (بواقع 79.7‏%) لديها معدلات منخفضة للإصابة بالمرض. ونصت السياسات على وجوب إجراء فحص للأطفال دون عمر 5 سنوات العرضة للاتصال مع أفراد الأسرة المصابين بمرض السل، وذلك في 25 بلدًا (بنسبة 83.3‏%) تعاني من معدلات مرتفعة للإصابة بالمرض وكذلك في 28 بلدًا (بنسبة 41.2‏%) لديها معدلات منخفضة. واقتصرت التوصيات في معظم البلدان التي تعاني من معدلات مرتفعة للإصابة بالمرض على مجرد إجراء فحص قائم على أعراض الإصابة قبل العلاج، في حين أن البلدان التي لديها معدلات منخفضة للإصابة تلتزم بإجراء الاختبارات قبل العلاج. ولا تتوافق سياسات بعض البلدان التي لديها معدلات منخفضة للإصابة مع توصيات منظمة الصحة العالمية؛ حيث أوصت تسعة بلدان (‏13.2%) المسافرين إلى ابلدان التي تعاني من ارتفاع معدلات الإصابة بمرض السل بالحصول على علاج وقائي للمرض، في حين أوصت عشرة بلدان (‏14.7%) بالحصول على العلاج الوقائي للمرضى الذين يخضعون لجراحة في البطن.

**الاستنتاج:**

يقود الافتقار إلى أدلة قوية بشأن بعض جوانب التعامل مع عدوى السل الكامنة إلى اعتماد سياسات وطنية متفاوتة بصورة كبيرة. ويسلط ذلك الضوء على الحاجة إلى النهوض بالبحث وتطوير سياسات واضحة وقابلة للتنفيذ وقائمة على الأدلة من جانب منظمة الصحة العالمية.

## 摘要

**目标:**

回顾结核病低负担国家和结核病高负担国家在潜伏性结核感染上的管理政策。

**方法:**

我们根据世界卫生组织 (WHO) 的标准将向 WHO 全球结核病项目汇报数据的国家划分为结核病低负担国家和结核病高负担国家。我们通过在线搜索、政府网站、WHO 国家办事处和与项目管理者的个人沟通，确定了关于潜伏性结核感染管理的国家政策文件。我们进行了描述性分析，重点关注该国政策与 WHO 政策建议的差距和偏差。

**结果:**

在 113 个结核病低负担国家和 35 个结核病高负担国家中，我们分别获得了 68 个国家和 30 个国家的结核病或人类免疫缺陷病毒 (HIV) 相关结核病的文件。在 29 个结核病高负担国家 (96.7%) 和 54 个 (79.7%) 结核病低负担国家的指导方针中，建议对 HIV 感染者进行潜伏性结核感染的筛查和治疗。25 个 (83.3%) 结核病高负担国家和 28 个 (41.2%) 结核病低负担国家的政策规定，对 5 岁以下、家中有结核病接触的孩子进行筛查。在大多数高负担国家，建议在治疗前单独进行症状筛查，而在全部低负担国家，则在治疗之前进行检测。一些低负担国家的政策不符合 WHO 建议：其中有 9 项 （13.2％） 政策建议对准备去结核病高负担国家的游客进行结核病预防治疗，10 项 （14.7％） 建议患者接受腹部手术。

**结论:**

潜伏性结核感染管理的某些方面缺乏确凿依据，导致国家政策方面有很大差异。这突出了对推动研究，制定清晰、可执行和以证据为基础的 WHO 政策的需求。

## Резюме

**Цель:**

Провести обзор политики по борьбе с латентной туберкулезной инфекцией в странах с низким и высоким бременем туберкулеза.

**Методы:**

На основе критериев ВОЗ авторы разделили страны, предоставляющие данные для Глобальной программы борьбы с туберкулезом Всемирной организации здравоохранения (ВОЗ), на несущие низкое и высокое бремя туберкулеза. Авторы определили документы национальной политики по борьбе с латентным туберкулезом посредством онлайн-поиска, правительственных веб-сайтов, региональных бюро ВОЗ и личного общения с руководителями программ. Был проведен описательный анализ с упором на пробелы и отклонения от рекомендаций ВОЗ в политике.

**Результаты:**

Были получены документы от 68 из 113 стран с низким бременем и от 30 из 35 стран с самым высоким бременем туберкулеза или вируса иммунодефицита человека (ВИЧ), связанного с туберкулезом. Скрининговое обследование и лечение латентной туберкулезной инфекции у людей с ВИЧ были рекомендованы в руководящих принципах 29 стран (96,7%) с высоким бременем и 54 стран (79,7%) с низким бременем туберкулеза. Скрининговое обследование детей в возрасте до 5 лет, находящихся в контакте с больными туберкулезом в бытовых условиях, проводился в рамках политики 25 стран (83,3%) с высоким бременем и 28 стран (41,2%) с низким бременем. В большинстве стран с высоким бременем туберкулеза перед началом лечения рекомендовано только скрининговое обследование симптомов, тогда как во всех странах с низким бременем проводится тестирование. Политика некоторых стран с низким бременем не соответствовала рекомендациям ВОЗ: в девяти странах (13,2%) рекомендована профилактика туберкулеза для путешественников, выезжающих в страны с высоким бременем, и в 10 (14,7%) — для пациентов, перенесших абдоминальное хирургическое вмешательство.

**Вывод:**

Отсутствие убедительных доказательств некоторых аспектов борьбы с латентной туберкулезной инфекцией приводит к значительным различиям в национальной политике. Это подчеркивает необходимость продвижения исследований и разработки четкой, реализуемой и основанной на фактических данных политики ВОЗ.

## Introduction

Tuberculosis is currently the leading infectious cause of death worldwide. The World Health Organization (WHO) End Tuberculosis strategy aims to substantially reduce tuberculosis incidence by 90% and mortality by 95% compared with the 2015 baselines of 142 cases per 100 000 population and 5.3 to 19 cases per 100 000 (depending on human immunodeficiency virus (HIV) status), respectively^1^^,^^2^ Achieving this goal requires successful management of latent tuberculosis infection, which serves as a reservoir for new tuberculosis cases.^3^ In high-income countries which already have a low incidence of tuberculosis, management of latent infection can contribute to elimination of the disease.^4^ A review of treatment regimens found that treatment of latent tuberculosis can reduce the risk of disease reactivation by 60% to 90%.^5^ A recent randomized controlled trial in a high tuberculosis burden country showed that the benefits of preventive treatment in people living with HIV can last for more than 5 years.^6^^,^^7^

The WHO recommends tailored latent tuberculosis infection management based on tuberculosis burden and resource availability.^8^ Systematic testing and treatment for latent infection is strongly recommended for people living with HIV and for children younger than 5 years who are household contacts of a pulmonary tuberculosis case, regardless of the country’s background tuberculosis burden or resource availability.^9^^,^^10^ In upper-middle or high-income countries, depending on low tuberculosis burden and availability of resources, systematic testing and treatment of latent tuberculosis is strongly recommended for certain other risk groups: adult household contacts of pulmonary tuberculosis cases; patients with silicosis; patients initiating anti-tumour necrosis factor treatment; patients on dialysis; and organ transplant recipients.^11^^,^^12^

Despite some progress, particularly over the last decade, the scale-up of tuberculosis preventive treatment remains suboptimal globally. The 161 740 children started on tuberculosis preventive treatment in 2016 represented only 13% of the 1.3 million children estimated to be eligible for treatment.^1^ The total number of people living with HIV who were started on tuberculosis preventive treatment in 2016 was at least 1.3 million.^1^ Data for other risk groups are not available or very limited.

Barriers to scale-up of tuberculosis preventive treatment include the absence of national policies and a lack of monitoring and evaluation systems.^13^ Here we review national policy documents to identify differences in programmatic management of latent tuberculosis infection in high- and low-burden countries.

## Methods

**Fig. 1:**
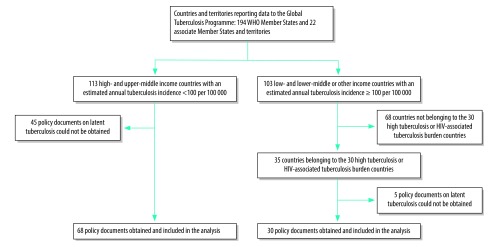
Selection of countries for the review of national policies on management of latent tuberculosis infection

The baseline for this descriptive policy review was the 216 countries and territories reporting data to the WHO Global Tuberculosis Programme (194 Member States and 22 associate Member States and territories). Based on the current WHO approach^11^ we divided countries into two groups: low burden and high burden. We defined low-burden countries as upper-middle or high-income countries with an estimated annual tuberculosis incidence of less than 100 cases per 100 000 population.^11^ High-burden countries were low- to lower-middle-income or other income countries with annual tuberculosis incidence of 100 or more cases per 100 000. Among the high-burden countries, we focused on the top 30 countries in terms of high burden of tuberculosis (both in terms of number of cases and incidence) and on the top 30 countries in terms of high burden of HIV-associated tuberculosis. These countries account for most of the global burden of tuberculosis (9.1 out of 10.4 million cases, 88%) and HIV-associated tuberculosis (0.91 out of 1.03 million, 88%).^1^ Because many countries feature in the top 30 of both lists, this produced a list of 35 target countries. Our approach resulted in a target of 148 countries (113 low-burden countries and 35 high-burden countries) for the study (Fig. 1).

HIV: human immunodeficiency virus.

We aimed to analyse each country’s or territory’s national guidelines on the management of tuberculosis, HIV, paediatric tuberculosis, latent tuberculosis infection and HIV-associated tuberculosis, and standard national operating procedures for tuberculosis. We obtained documents by contacting WHO country offices or national programme managers or by downloading them from the official website of the ministry of health or other national health organization.

We selected the information to be extracted a priori based on WHO recommendations for the management of latent tuberculosis infection (Table 1 and Table 2).^9^^–^^11^ We collected information on the following: (i) at-risk populations targeted; (ii) recommended tests for latent tuberculosis infection; (iii) diagnostic algorithms to exclude active tuberculosis before starting treatment for latent tuberculosis infection; (iv) treatment regimens for latent tuberculosis infection; and (v) presence of monitoring and evaluation systems for the management of latent tuberculosis infection. For high-burden countries, we focused the review only on people living with HIV and children younger than 5 years who have household contact with a tuberculosis case. One researcher collected and entered the data for all low-burden countries and another researcher for all high-burden countries using data extraction forms developed for the study.

**Table 1:** World Health Organization recommendations for the management of latent tuberculosis infection in low and high tuberculosis burden countries, October 2017

Tuberculosis burden classification	Risk groups defined	Testing recommendations	Diagnostic algorithms to exclude active tuberculosis	Treatment recommendations
Low-burden countries	Strong recommendation: people living with HIV; adult and child household contacts of pulmonary tuberculosis cases; treatment with anti-tumour necrosis factor; organ transplantation; silicosis; end-stage renal disease Conditional recommendation: health-care workers; prisoners; immigrants from high-burden countries; illicit drug users; homeless people	Tuberculin skin test or interferon-gamma release assay	Symptomatic screening plus chest X-ray	6 months daily isoniazid; or 9 months daily isoniazid; or 3 months weekly rifapentine plus isoniazid; or 3–4 months daily isoniazid plus rifampicin; or 3–4 months daily rifampicin
High-burden countries	People living with HIV; children aged < 5 years; household contacts of pulmonary tuberculosis cases	Tuberculin skin test or interferon-gamma release assay not required. Tuberculin skin test encouraged for people living with HIV	Symptomatic screening alone	6 months daily isoniazid

HIV: human immunodeficiency virus.

Sources: World Health Organization (WHO*) Guidelines on the management of latent tuberculosis infection*.^11^ *WHO Recommendations for investigating contacts of persons with infectious tuberculosis in low- and middle-income countries*.^9^ *WHO Guidelines for intensified case-finding and isoniazid preventative therapy for people living with HIV in resource-constrained settings*.^10^

**Table 2:** Definitions of symptoms to exclude active tuberculosis before providing tuberculosis preventive treatment to adults and children living with human immunodeficiency virus (HIV) in countries with the highest burdens of tuberculosis or HIV-associated tuberculosis

Country	Adults living with HIV			Children living with HIV	
	Symptoms defined by WHO	Additional symptoms or findings		Symptoms defined by WHO	Additional symptoms or findings
Angola	Current cough; fever; night sweats; weight loss	N/A		Current cough; fever; weight loss or poor weight gain	Chest X-ray findings suggestive of tuberculosis
Bangladesh	N/A	N/A		Current cough; fever; contact history with a tuberculosis case; weight loss or poor weight gain	Fatigue, lethargy, neck mass, wheeze, ascites
Botswana	N/A	N/A		N/A	N/A
Cambodia	Current cough; fever; night sweats	Fatigue; lethargy; wheeze; neck mass; abdominal mass; ascites		Current cough; fever; contact history with a tuberculosis case; weight loss or poor weight gain	Failure to thrive, enlarged lymph nodes
Cameroon	Current cough; fever; night sweats; weight loss	N/A		Current cough; fever; weight loss or poor weight gain	N/A
Central African Republic	Current cough; fever; night sweats; weight loss	N/A		Current cough; fever; contact history with a tuberculosis case; weight loss or poor weight gain	N/A
Democratic Republic of the Congo	Current cough; fever; night sweats; weight loss	N/A		Current cough; fever; contact history with a tuberculosis case; weight loss or poor weight gain	N/A
Ethiopia	Current cough; fever; night sweats; weight loss	N/A		Current cough; fever; contact history with a tuberculosis case; weight loss or poor weight gain	N/A
Ghana	N/A	N/A		Current cough; fever; contact history with a tuberculosis case; weight loss or poor weight gain	Fatigue, lethargy, neck swelling, wheeze
India	Current cough; fever; night sweats; weight loss	N/A		N/A	N/A
Indonesia	Current cough; fever; night sweats; weight loss	Signs of extrapulmonary tuberculosis		N/A	N/A
Kenya	Current cough; fever; night sweats; weight loss	N/A		Current cough; fever; contact history with a tuberculosis case; weight loss or poor weight gain	Lethargy, less playful than usual
Lesotho	Current cough; fever; night sweats; weight loss	N/A		Current cough; fever; contact history with a tuberculosis case; weight loss or poor weight gain	N/A
Malawi	Current cough; fever; night sweats; weight loss	N/A		Current cough; fever	Failure to thrive, night sweats, malnutrition
Mozambique	N/A	N/A		Current cough; fever; weight loss or poor weight gain	N/A
Myanmar	Current cough; fever; night sweats; weight loss	N/A		Current cough; fever; contact history with a tuberculosis case; weight loss or poor weight gain	N/A
Namibia	Current cough; fever; night sweats; weight loss	Chest pain; shortness of breath; haemoptysis; loss of appetite; diarrhoea; fatigue; enlarged lymph nodes		Current cough; fever; contact history with a tuberculosis case; weight loss or poor weight gain	Enlarged lymph nodes
Nigeria	Current cough; fever; night sweats; weight loss	N/A		N/A	N/A
Pakistan	Current cough; fever; night sweats; weight loss	N/A		Current cough; fever; contact history with a tuberculosis case; weight loss or poor weight gain	N/A
Papua New Guinea	Current cough; night sweats; weight loss	N/A		N/A	N/A
Philippines	Current cough; fever; night sweats; weight loss	N/A		N/A	N/A
Sierra Leone	Current cough; fever; night sweats; weight loss	N/A		N/A	N/A
South Africa	Current cough; fever; night sweats; weight loss	N/A		Current cough; fever; contact history with a tuberculosis case; weight loss or poor weight gain	Fatigue
Swaziland	Current cough; fever; night sweats; weight loss	N/A		Current cough; fever; contact history with a tuberculosis case; weight loss or poor weight gain	N/A
Thailand	N/A	N/A		N/A	N/A
Uganda	Current cough; fever; night sweats; weight loss	N/A		N/A	N/A
United Republic of Tanzania	Current cough; fever; night sweats; weight loss	N/A		Fever; contact history with a tuberculosis case; weight loss or poor weight gain	N/A
Viet Nam	Current cough; fever; night sweats; weight loss	N/A		N/A	N/A
Zambia	Current cough; fever; night sweats; weight loss	N/A		Current cough; fever; contact history with a tuberculosis case; weight loss or poor weight gain	N/A
Zimbabwe	Current cough; fever; night sweats; weight loss	Haemoptysis		N/A	N/A

HIV: human immunodeficiency virus; N/A: data not available; WHO: World Health Organization.

Notes: WHO recommended four-symptom algorithm for people living with HIV includes: current cough, fever, night sweats and weight loss for adults and current cough, fever, poor weight gain and contact history with a tuberculosis case for children.^10^

Statistical analysis of the data was performed using GraphPad Prism (GraphPad Software Inc., La Jolla, United States of America) and STATA (Stata Corp LLC, College Station, USA) software. Where percentages are indicated, binary indicators (0,1) were created for the absence or presence of each policy item extracted. The means of those binary indicators corresponded to the percentage of countries addressing each policy item. The number and percentage of countries addressing each policy item were calculated and presented.

## Results

### Results of search

We obtained and analysed copies of policy documents from 98 countries (Table 3; available at: http://www.who.int/bulletin/volumes/96/3/17-199414). For high-burden countries, we obtained guidelines from 30 of 35 (85.7%) high-burden countries. We were unable to retrieve any national policies pertaining to latent tuberculosis infection for Chad, Democratic People's Republic of Korea, Guinea Bissau, Liberia and Congo. For guidelines obtained, publication year ranged between 2007 and 2016. We also included one draft guideline under review.

**Table 3:** List of countries and territories included in the review of national policies on the management of latent tuberculosis infection and World Health Organization recommendations on tuberculosis

Country or territory	WHO region	Country group	Data source	
**High tuberculosis burden countries**				
Angola	AFR	High tuberculosis and HIV-associated tuberculosis burden	*Nota tecnica sobre as mundancas no diagnostico e tretamento da infeccao pelo VIH e SIDA em Angola para adultos, gestantes, adolescentes e criancas*. Luanda: Ministerio da Saude, Insituto Nacional de Luta contra a SIDA; 2014. *Rotocolo para avaliação e seguimento de enfermagem aos pacientes VIH^+^*. Luanda: Ministerio da Saude, Insituto Nacional de Luta contra a SIDA; 2014.	
Bangladesh	SEA	High tuberculosis burden	*National guidelines for the management of tuberculosis in children*. Dhaka: National Tuberculosis Control Programme, Ministry of Health and Family Welfare; 2012. *National guidelines of antiretroviral therapy, Bangladesh*. Dhaka: National AIDS/STD Programme, Directorate General Health Services, Ministry of Health and Family Welfare; 2011.	
Botswana	AFR	High HIV-associated tuberculosis burden	*Botswana national HIV and AIDS treatment guidelines*. Gaborone: Ministry of Health; 2012. *Tuberculosis/HIV policy guidelines*. Gaborone: Ministry of Health; 2011.	
Cambodia	WPR	High tuberculosis burden	*National guidelines for diagnosis and treatment of tuberculosis in children*. Phnom Penh: National Centre for Tuberculosis and Leprosy Control, Ministry of Health; 2008. *Standard operating procedures for implementing the three I's in continuum of care settings*. Phnom Penh: National Centre for HIV/AIDS Dermatology and STD and National Centre for Tuberculosis and Leprosy Control; 2010.	
Cameroon	AFR	High HIV-associated tuberculosis burden	*Directives nationales de prevention et de prise en charge du VIH au Cameroun*. Yaoundé: Ministère de la Santé; 2015. *Guide technique pour les personnels de Sant*é. Yaoundé: Programme National de Lutte contre la Tuberculose, Ministère de la Santé; 2012.	
Central African Republic	AFR	High tuberculosis and HIV-associated tuberculosis burden	*Guide de prise en charge de la tuberculose de l’adulte*. Bangui: Programme National de Lutte contre la Tuberculose. Ministère de la Santé de l’Hygiene Publique et de la Population; 2016. *Guide de prise en charge de la tuberculose de l’enfant*. Bangui: Programme National de Lutte Contre la Ministère de la Santé de l’Hygiene Publique et de la Population; 2016.	
Democratic Republic of the Congo	AFR	High tuberculosis and HIV-associated tuberculosis burden	*Guide national de prise en charge de l’infection a VIH en RDC*. Kinshasa: Programme National de Lutte Contre le VIH/SIDA et les ISTs PNLS; 2013. *Guide de prise en charge de la co-infection VIH-tuberculose dans la zone de sant*é. Kinshasa: Ministère de la Santé Public; 2012.	
Ethiopia	AFR	High tuberculosis and HIV-associated tuberculosis burden	*Guidelines for clinical programmatic management of tuberculosis, tuberculosis/HIV and leprosy in Ethiopia*. Ministry of Health; 2013. *National guidelines for comprehensive HIV prevention, care and treatment*. Addis Ababa: Ministry of Health; 2014. *National childhood tuberculosis implementation guide*. Addis Ababa: Ministry of Health; 2015.	
Ghana	AFR	High HIV-associated tuberculosis burden	*Guidelines for diagnosis and management of tuberculosis in children*. Accra: Ghana Health Service; 2012. *Guidelines for the clinical management of tuberculosis and HIV co-infection*. Accra: Ghana Health Service; 2007. *Guidelines for antiretroviral therapy in Ghana.* Accra: National HIV/AIDS/STI Control Programme, Ministry of Health and Ghana Health Service; 2008.	
India	SEA	High tuberculosis and HIV-associated tuberculosis burden	*National guidelines on diagnosis and treatment of paediatric tuberculosis*. New Delhi: Central Tuberculosis Division, Directorate General of Health Services, Ministry of Health and Family Welfare; 2012. *DOTS-plus guidelines*. New Delhi: Revised National Tuberculosis Control Programme; 2010. *Standards for tuberculosis care in India*. New Delhi: Central Tuberculosis Division, Directorate General of Health Services, Ministry of Health and Family Welfare; 2014.	
Indonesia	SEA	High tuberculosis and HIV-associated tuberculosis burden	*National policy on tuberculosis/HIV collaboration* [translated]. Jakarta: Ministry of Health; 2015. *Technical guidelines on management of tuberculosis in children* [translated]. Jakarta: Ministry of Health; 2013.	
Kenya	AFR	High tuberculosis and HIV-associated tuberculosis burden	*National isoniazid preventative therapy standard operating procedure*. Nairobi: Ministry of Health; 2015. *Guidelines for management of tuberculosis and leprosy in Kenya*. Nairobi: Ministry of Health; 2013.	
Lesotho	AFR	High tuberculosis and HIV-associated tuberculosis burden	*National guidelines for the three I’s (isoniazid preventive therapy (IPT) intensified case finding (ICF) and infection control (IC).* Maseru: Government of Lesotho; 2011. *National tuberculosis programme policy and manual*. Maseru: Government of Lesotho; 2007.	
Malawi	AFR	High HIV-associated tuberculosis burden	*National tuberculosis control programme manual*. Lilongwe: Ministry of Health; 2012. *Clinical management of HIV in children and adults*. Lilongwe: Ministry of Health; 2014.	
Mozambique	AFR	High tuberculosis and HIV-associated tuberculosis burden	*Guia de tratamento antiretroviral e infecções oportunistas no adulto, adolescente grávida e criança*. Maputo: Ministro da Saúde; 2014.	
Myanmar	SEA	High tuberculosis and HIV-associated tuberculosis burden	*Guidelines for the programmatic management of tuberculosis/HIV in Myanmar*. Naypyidaw: National Tuberculosis Programme and National AIDS Programme; 2015.	
Namibia	AFR	High tuberculosis and HIV-associated tuberculosis burden	*National guidelines for the management of tuberculosis*. Windhoek: Ministry of Health and Social Services; 2011. *National guidelines for HIV prevention treatment and care*. Windhoek: Ministry of Health and Social Services; 2014.	
Nigeria	AFR	High tuberculosis and HIV-associated tuberculosis burden	*National tuberculosis and leprosy control programme – worker’s manual*. Abuja: Department of Public Health, Federal Ministry of Health; 2010.	
Pakistan	EMR	High tuberculosis burden	*National guidelines for the control of tuberculosis in Pakistan*. Islamabad: National TB Control Programme, Ministry of National Health Services, Regulation and Coordination; 2015.	
Papua New Guinea	WPR	High tuberculosis and HIV-associated tuberculosis burden	*National tuberculosis management protocol*. Port Moresby: Department of Health, Disease Control Branch, National Tuberculosis Programme; 2011. *Guidelines for HIV care and treatment in Papua New Guinea*. Port Moresby: Department of Health; 2009.	
Philippines	WPR	High tuberculosis burden	*National tuberculosis control programme: manual of procedures, 5th edition*. Manila: Department of Health; 2014.	
Sierra Leone	AFR	High tuberculosis burden	*Tuberculosis treatment guidelines* [draft]. Freetown: Ministry of Health and Sanitation; 2016. *National antiretroviral treatment guidelines*. Freetown: Ministry of Health and Sanitation; 2015.	
South Africa	AFR	High tuberculosis and HIV-associated tuberculosis burden	*Guidelines for the management of tuberculosis in children*. Pretoria: Department of Health, 2013. *National tuberculosis management guidelines*. Pretoria: Department of Health; 2014. *Guidelines for tuberculosis preventive therapy among HIV infected individuals in South Africa*. Pretoria: Department of Health; 2010. *National consolidated guidelines for the prevention of mother-to-child transmission of HIV (PMTCT) and the management of HIV in children, adolescents and adults*. Pretoria: Department of Health; 2014.	
Swaziland	AFR	High HIV-associated tuberculosis burden	*Swaziland integrated HIV management guidelines*. Mbabane: Ministry of Health; 2015. *National policy guidelines on TB/HIV collaborative activities*. Mbabane: Ministry of Health; 2015.	
Thailand	SEA	High tuberculosis and HIV-associated tuberculosis burden	*Clinical practices guidelines of tuberculosis treatment in adults*. Nonthaburi: Department of Tuberculosis, Department of Disease Control, Ministry of Public Health; 2013.	
Uganda	AFR	High HIV-associated tuberculosis burden	*Ministry of health manual of the national tuberculosis and leprosy programme*. Kampala: Ministry of Health; 2010. *The integrated national guidelines on antiretroviral therapy prevention of mother to child transmission of HIV infant and young child feeding*. Kampala: Ministry of Health; 2012.	
United Republic of Tanzania	AFR	High tuberculosis and HIV-associated tuberculosis burden	*National guidelines for the management of tuberculosis in children*. Dar es Salaam: Ministry of Health and Social Welfare; 2013. *Manual for the management of tuberculosis and leprosy*. Dar es Salaam: Ministry of Health and Social Welfare; 2013. *National policy guidelines for collaborative TB/HIV activities*. Dar es Salaam: Ministry of Health and Social Welfare; 2008. *National guidelines for the management of HIV and AIDS*. Dar es Salaam: Ministry of Health and Social Welfare; 2012.		
Viet Nam	WPR	High tuberculosis burden		*Diagnosis, treatment, and prevention of tuberculosis* [translated]. Hanoi: Ministry of Health; 2015. *Guidelines for HIV/AIDS diagnosis and treatment*. Hanoi: Ministry of Health; 2014. *Collaborative protocol for TB/HIV diagnosis, treatment and case management*. Hanoi: Ministry of Health; 2007.
Zambia	AFR	High tuberculosis and HIV-associated tuberculosis burden		*National guidelines on management of tuberculosis in children*. Lusaka: Ministry of Health, Division of Leprosy, Tuberculosis and Lung Disease; 2013. *Zambia consolidated guidelines for treatment and prevention of HIV infection*. Lusaka: Ministry of Health and Ministry of Community Development, Mother and Child Health; 2014.
Zimbabwe	AFR	High tuberculosis and HIV-associated tuberculosis burden		*National TB guidelines, 4th edition*. Harare: National Tuberculosis Control Programme, Ministry of Health and Child Welfare; 2010. *National guidelines for TB/HIV co-management*. Harare: National HIV/AIDS and Tuberculosis Control Programmes. Ministry of Health and Child Welfare; 2014.
**Low-tuberculosis burden countries**				
Algeria	AFR	Low-tuberculosis burden		*Measure de prevention pour les sujets contact. Chapitre VI.* In: *La prevention de la tuberculose.* Alger: Ministère de la Santé, de la Population et de la Réforme Hospitalière; 2011
American Samoa	WPR	Low-tuberculosis burden		*Latent tuberculosis infection: a guide for primary care providers*. Atlanta: United States Centers for Disease Control and Prevention; 2013.
Antigua and Barbuda	AMR	Low tuberculosis burden		*Caribbean guidelines for the prevention, treatment, care and control of tuberculosis and HIV/TB*. Washington: Caribbean HIV/AIDS Regional Training Network; 2010.
Argentina	AMR	Low tuberculosis burden		*Programa nacional de control de la tuberculosis*. *Normas técnicas*. Buenos Aires: Ministerio de Salud de la Nación; 2013.
Aruba	AMR	Low tuberculosis burden		*Caribbean guidelines for the prevention, treatment, care and control of tuberculosis and HIV/tuberculosis*. Washington: Caribbean HIV/AIDS Regional Training Network; 2010.
Australia	WPR	Low tuberculosis burden		*CDNA national guidelines for the public health management of tuberculosis*. Canberra: Department of Health; 2013.
Austria	EUR	Low tuberculosis burden		*Österreichische leitlinie zurtuberkulose – umgebungsuntersuchung*. Vienna: Bundesministerium für Gesundheit; 2013.
Bahamas	AMR	Low tuberculosis burden		*Tuberculosis contro*l [Internet]. Nassau: Ministry of Health; 2011.
Barbados	AMR	Low tuberculosis burden		*Caribbean guidelines for the prevention, treatment, care and control of tuberculosis and HIV/TB*. Washington: Caribbean HIV/AIDS Regional Training Network; 2010.
Belgium	EUR	Low tuberculosis burden		*Recommendations concernant le depistage cible et le traitement de l'infection tuberculeuse latente*. Brussels: Fondation Contre Les Affections Respiratoires et Pour L'Education a La Sante; 2003.
Belize	AMR	Low tuberculosis burden		*Caribbean guidelines for the prevention, treatment, care and control of tuberculosis and HIV/TB*. Washington: Caribbean HIV/AIDS Regional Training Network; 2010.
Bermuda	AMR	Low tuberculosis burden		*Caribbean guidelines for the prevention, treatment, care and control of tuberculosis and HIV/TB*. Washington: Caribbean HIV/AIDS Regional Training Network; 2010.
Brazil	AMR	Low tuberculosis burden		*Manual de recomendacoes para o controle da tuberculose no Brazil*. Brasília: Ministerio da Salude; 2011.
Brunei Darussalam	WPR	Low tuberculosis burden		*Guidelines for tuberculosis control in Brunei Darussalam.* Bandar Seri Begawan: Ministry of Health; 2013.
Canada	AMR	Low tuberculosis burden		*Canadian tuberculosis standards*, *7th edition*. Ottawa: Public Health Agency of Canada; 2014.
Chile	AMR	Low tuberculosis burden		*Normas tecnicas para el control y la eliminacion de la tuberculosis*. Santiago: Programa Nacional para el Control y la Eliminacion de la Tuberculosis; 2014.
China, Hong Kong Special Administrative Region	WPR	Low tuberculosis burden		*Guidelines on targeted tuberculin testing and treatment of latent tuberculosis infection*. Hong Kong: Tuberculosis and Chest Service; 2015 (last update on 31 March 2015).
Colombia	AMR	Low tuberculosis burden		*Guías de promoción de la salud y prevención de enfermedades en la salud pública. Guía II: Guía de atención de la tuberculosis pulmonar y extrapulmonar.* Bogotá: Programa de Apoyo a la Reforma de Salud/PARS, Ministerio de la Protección Social; 2005.
Costa Rica	AMR	Low tuberculosis burden		*Manual de normas de atencion y vigilancia para el control de la tuberculosis*. San José: Programa Nacional para el Control de la Tuberculosis; 2003.
Cyprus	EUR	Low tuberculosis burden		*Prolypsi tis metadosis fymatiosis se xorous paroxhs yperesion ygeias*. [Prevention of the transmission of tuberculosis to health-care facilities.] Nicosia: Ministry of Health; 2015.
Czechia	EUR	Low tuberculosis burden		*Vyhláška 473/2008 Sb: Ministerstva zdravotnictví ze dne 17 prosince 2008 o systému epidemiologické bdělosti pro vybrané infekce*. [Declaration 473/2008 Sb by the Ministry of Health of 17 December 2008 on an epidemiological alert system for selected infections.] Prague: Ministry of Health; 2008.
Dominican Republic	AMR	Low tuberculosis burden		*Reglamento tecnico para la prevencion y el control de la tuberculosis*. Santo Domingo: Ministerio de Salud Publica; 2014. *Normas nacionales para el control de la tuberculosis en Republica Dominicana*. *Serie de normas nacionales no. 16*. Santo Domingo D.N.: Ecretaria de Estado de Salud Publica y Asistencia Social, Programa Nacional de Control de la Tuberculosis; 2003.
Ecuador	AMR	Low tuberculosis burden		*Manual de normas y procedimientos para el control de la tuberculosis en Ecuador.* Quito: Ministerio de Salud Publica; 2010.
Fiji	WPR	Low tuberculosis burden		*Tuberculosis guideline*. Suva: National Tuberculosis Programme; 2011.
Finland	EUR	Low tuberculosis burden		*Suositus tuberkuloosin kontaktiselvityksen toteuttamiseksi*. Helsinki: Terveyden ja hyvinvoinnin laitos; 2011.
France	EUR	Low tuberculosis burden		*Avis du conseil superieur d'hygiene publique de France section maladies transmissibles. Relatif au traitment de la tuberculose-infection*. Paris: Direction General de la Santé. Ministère de la Santé, de la Famille et des Personnes handicapées; 2003.
French Polynesia	WPR	Low tuberculosis burden		*Guide pratique a l'intention de professionels de santé*. Papeete: Direction de la Santé, Département des Programmes de Prévention, Programme Contre la Tuberculose; 2011.
Germany	EUR	Low tuberculosis burden		*New recommendations for contact tracing in tuberculosis.* Stuttgart: German Central Committee against Tuberculosis; 2011.
Greece	EUR	Low tuberculosis burden		*Fymatiosi: apo ti diagnosi sti therapia. [Tuberculosis: from diagnosis to therapies.]* Athens: Elliniki Pneumologiki Etairia; 2012.
Grenada	AMR	Low tuberculosis burden		*Caribbean guidelines for the prevention, treatment, care and control of tuberculosis and HIV/tuberculosis*. Washington: Caribbean HIV/AIDS Regional Training Network; 2010.
Ireland	EUR	Low tuberculosis burden		*Guidelines on the prevention and control of tuberculosis in Ireland*. Dublin: Health Protection and Surveillance Centre; 2010 [amended 2014].
Israel	EUR	Low tuberculosis burden		D. Chemtob, A. Leventhal,Y. Berlowitz,D. Weiler-Ravell. The new national tuberculosis control programme in Israel, a country of high immigration. *Int J Tuberc Lung Dis*. 2003 Sep;7(9):828–36. PMID:12971665
Jamaica	AMR	Low tuberculosis burden		*Caribbean guidelines for the prevention, treatment, care and control of tuberculosis and HIV/tuberculosis*. Washington: Caribbean HIV/AIDS Regional Training Network; 2010.
Japan	WPR	Low tuberculosis burden		*Treatment guidelines for latent tuberculosis infection*. Tokyo: Prevention Committee and the Treatment Committee of the Japanese Society for Tuberculosis; 2013.
Malaysia	WPR	Low tuberculosis burden		*Management of tuberculosis, 3rd edition*. Putrajaya: Malaysia Health Technology Assessment Section; 2012.
Malta	EUR	Low tuberculosis burden		*Prevention, control and management of tuberculosis: a national strategy for Malta*. Msida: Infectious Disease Prevention and Control Unit, Ministry of Health; 2012.
Mexico	AMR	Low tuberculosis burden		*Norma oficial Mexicana NOM-006-SSA2–2013, para la prevención y control de la tuberculosis*. Mexico City: Secretaría de Salud; 2013.
Netherlands	EUR	Low tuberculosis burden		*Richtlijn behandeling latente tuberculose-infectie*. The Hague: KNCV Tuberculosis Foundation; 2015.
New Zealand	WPR	Low tuberculosis burden		*Guidelines for tuberculosis control in New Zealand 2010. Chapter 8.* Wellington: Ministry of Health; 2010.
Norway	EUR	Low tuberculosis burden		*Tuberkuloseveilederen: 10. Forebyggende behandling av latent tuberkulose (LTB).* Oslo: Folkehelseinstituttet; 2010.
Oman	EMR	Low tuberculosis burden		*STOP tuberculosis. Manual of tuberculosis control programme*, *4th edition, April 2007*. Muscat: National Tuberculosis Control Programme Department of Communicable Disease Surveillance and Control, Directorate General of Health Affairs, Ministry of Health; 2007.
Palau	WPR	Low tuberculosis burden		*Latent tuberculosis infection: a guide for primary care providers*. Atlanta: United States Centers for Disease Control and Prevention; 2013.
Panama	AMR	Low tuberculosis burden		*Norma nacional para la prevencion y control de la tuberculosis*. Panama City: Ministerio de Salud; *;* 2015.
Poland	EUR	Low tuberculosis burden		*Tuberculosis manual: national tuberculosis programme guidelines*. Warsaw: National Tuberculosis and Lung Diseases Research Institute; 2001.
Portugal	EUR	Low tuberculosis burden		*Tratamento da tuberculose latente revisão das normas*. Lisbon: Sociedade Portuguesa de Pneumologia; 2014.
Puerto Rico	AMR	Low tuberculosis burden		*Latent tuberculosis infection: a guide for primary care providers*. Atlanta: United States Centers for Disease Control and Prevention; 2013.
Saint Kitts and Nevis	AMR	Low tuberculosis burden		*Caribbean guidelines for the prevention, treatment, care and control of tuberculosis and HIV/tuberculosis*. Washington: Caribbean HIV/AIDS Regional Training Network; 2010.
Saint Lucia	AMR	Low tuberculosis burden		*Caribbean guidelines for the prevention, treatment, care and control of tuberculosis and HIV/tuberculosis*. Washington: Caribbean HIV/AIDS Regional Training Network; 2010.
Saint Vincent and the Grenadines	AMR	Low tuberculosis burden		*Caribbean guidelines for the prevention, treatment, care and control of tuberculosis and HIV/tuberculosis*. Washington: Caribbean HIV/AIDS Regional Training Network; 2010.
Saudi Arabia	EMR	Low tuberculosis burden		Saudi guidelines for testing and treatment of latent tuberculosis infection. 2010. Joint statement of the Saudi Thoracic Society, the Saudi Society of Medical Microbiology and Infectious Diseases, the Saudi Association of Public Health, and the Society of Family and Community Medicine. *Ann Saudi Med*. 2010 Jan-Feb; 30(1): 38–49.
Serbia	EUR	Low tuberculosis burden		*Guidelines for examining persons in contact with tuberculosis, latent tuberculosis and chemoprophylaxis. Project “tuberculosis control in Serbia”* [translated title]. Belgrade: Ministry of Health; 2011.
Seychelles	AFR	Low tuberculosis burden		*National tuberculosis programme: clinical diagnosis and management of tuberculosis, and measures for its prevention and control*. Mont Fleuri: Communicable Disease Control Unit Seychelles Hospital; 2013.
Singapore	WPR	Low tuberculosis burden		*A guide on infectious diseases of public health importance in Singapore*. Singapore: Ministry of Health; 2011.
Sint Maarten (Dutch part)	AMR	Low tuberculosis burden		*Caribbean guidelines for the prevention, treatment, care and control of tuberculosis and HIV/tuberculosis*. Washington: Caribbean HIV/AIDS Regional Training Network; 2010.
Spain	EUR	Low tuberculosis burden		*Guia de practica clinica sobre el diagnostico, el tratamiento y la prevencion de la tuberculosis. Guias de practica clinical en el SNS*. Madrid: Ministerio de Sanidad, Polica Social e Igualidad; 2010.
Suriname	AMR	Low tuberculosis burden		*Caribbean guidelines for the prevention, treatment, care and control of tuberculosis and HIV/tuberculosis*. Washington: Caribbean HIV/AIDS Regional Training Network; 2010.
Sweden	EUR	Low tuberculosis burden		*Rekommendationer för preventiva insatser mot tuberkulos: hälsokontroll, smittspårning och vaccination*. Stockholm: Socialstyrelsen; 2012.
Switzerland	EUR	Low tuberculosis burden		*Tuberculosis in Switzerland*. Bern: Swiss ￼Federal Office of Public Health; 2012.
The former Yugoslav Republic of Macedonia	EUR	Low tuberculosis burden		*Diagnosis and treatment of latent tuberculosis infection. Skopje*: PHI Institute for Lung Disease and Tuberculosis; 2009.
Trinidad and Tobago	AMR	Low tuberculosis burden		*Caribbean guidelines for the prevention, treatment, care and control of tuberculosis and HIV/tuberculosis*. Washington: Caribbean HIV/AIDS Regional Training Network; 2010.
Tunisia	EMR	Low tuberculosis burden		*Guide de prise en charge de la turbeculose en Tunisie*. Tunis: Direction des Soins de Santé de Base, République Tunisienne; 2014;
United Arab Emirates	EMR	Low tuberculosis burden		*Manual of tuberculosis control*. Abu Dhabi: National Tuberculosis Control Programme; Ministry of Health; 2010.
United Kingdom	EUR	Low tuberculosis burden		*Tuberculosis*. *NICE guidelines [NG33].* London: National Institute for Health and Care Excellence; 2016.
British Overseas Territory Turks and Caicos Islands	AMR	Low tuberculosis burden		*Caribbean guidelines for the prevention, treatment, care and control of tuberculosis and HIV/tuberculosis*. Washington: Caribbean HIV/AIDS Regional Training Network; 2010.
United States	AMR	Low tuberculosis burden		*Latent tuberculosis infection: a guide for primary care providers*. Atlanta: United States Centers for Disease Control and Prevention; 2013.
Uruguay	AMR	Low tuberculosis burden		Guía Nacional para el manejo de la Tuberculosis. Montevideo: Comision Honoraria para la Lucha Antituberculosa y Enfermedades Prevalentes; 2016.
United States Virgin Islands	AMR	Low tuberculosis burden		*Latent tuberculosis infection: a guide for primary care providers*. Atlanta: United States Centers for Disease Control and Prevention; 2013.
Venezuela (Bolivarian Republic of)	AMR	Low tuberculosis burden		*Directrices para el despistaje, diagnóstico y tratamiento de la tuberculosis en pacientes con indicación de terapias biológicas*. Caracas: Ministerio del Poder Popular para la Salud, Viceministerio de Redes de Salud Colectiva & Direccion General de Programas de Salud Coordinación Nacional de Salud Respiratoria; 2010.

AFR: African Region; AIDS: acquired immune deficiency syndrome; AMR: Region of the Americas; EMR: Eastern Mediterranean Region; EUR: European Region; HIV: human immunodeficiency virus; SEAR: South-East Asia Region; TB: tuberculosis; WHO: World Health Organization; WPR: Western Pacific Region.

For low-burden countries, we were able to obtain policy documents from 68 of 113 (60.2%) countries, with a publication year ranging from 2001 to 2015. The policy documents ranged from detailed policies focusing on latent tuberculosis infection to a brief mention of latent tuberculosis infection in a general tuberculosis policy.

### High-burden countries

#### Risk groups defined

Of the 30 high-burden countries for which guidelines were obtained, information on the management of latent tuberculosis infection among children with a household contact was available for 25 countries. In four countries the relevant tuberculosis guidelines could not be obtained and in one country the guidelines were written in local languages that we were not able to translate. All 25 countries followed WHO policy (Table 1) recommending treatment for children younger than 5 years with a household tuberculosis contact (Table 4); 17 of these specifically targeted contacts of smear-positive cases. India and Nigeria recommended preventive treatment for children under 6 years old with a household contact. No policy recommended preventive treatment for contacts of a multidrug-resistant tuberculosis case.

**Table 4:** Recommendations for management of latent tuberculosis infection in countries with the highest burdens of tuberculosis or HIV-associated tuberculosis

Indicator	Total no. of countries reviewed	No. (%) following recommendation
Guidelines identified on testing and treatment of tuberculosis, with or without HIV	35	30 (85.7)
**Latent tuberculosis infection treatment recommended for:**		
Children aged < 5 years with household tuberculosis contact		
Yes	30	25 (83.3)
Unknown^a^	30	5 (16.7)
People living with HIV		
Yes	30	29 (96.7)
No	30	1 (3.3)
**Recommended treatment regimens**		
6 months isoniazid monotherapy	30	18 (60.0)
6–9 months isoniazid monotherapy	30	6 (20.0)
Isoniazid monotherapy, other durations	30	5 (16.7)
6 months isoniazid and 3 months rifampicin	30	1 (3.3)
**Monitoring and evaluation indicators**		
Isoniazid preventive therapy for children aged < 5 years with household tuberculosis contact	30	4 (13.3)
Screening coverage among children aged < 5 years with household tuberculosis contact	30	7 (23.3)
Isoniazid preventive treatment coverage among HIV-infected people	30	18 (60.0)
Isoniazid preventive treatment reporting tool available	30	15 (50.0)
**Screening of children aged < 5 years old with household tuberculosis contact**		
Clinical examination only	30	24 (80.0)
Clinical examination and tuberculin skin test	30	1 (3.3)
Unknown	30	5 (16.7)
**Screening of HIV-infected people**		
Adults		
Clinical examination only	30	26 (86.7)
Clinical examination and tuberculin skin test	30	1 (3.3)
Not defined	30	3 (10.0)
Children aged > 12 months		
Clinical examination only	30	20 (66.7)
Clinical examination and tuberculin skin test	30	1 (3.3)
Clinical examination and chest X-ray	30	1 (3.3)
Not defined	30	8 (26.7)

HIV: human immunodeficiency virus.

^a^ We could not obtain any relevant treatment guidelines for child contacts and we found no recommendations in other available guidelines (4 countries) or the guidelines were written in a local language and we were unable to translate them with confidence (1 country).

For people living with HIV, 29 countries (96.7%) had recommendations on tuberculosis preventive treatment; only Ghana did not provide any recommendations.

#### Testing recommendations

For children younger than 5 years with a household contact, 24/25 (96.0%) of the countries analysed did not have recommendations for testing for latent tuberculosis before starting preventive treatment. Only in the Philippines was a tuberculin skin test recommended, with the option to provide preventive treatment without testing when testing was not available. To exclude active tuberculosis before treatment of latent tuberculosis, most countries (24/25, 96.0%) had a policy on symptomatic screening alone. Symptom-based algorithms to exclude active tuberculosis were defined in the guidelines of 12 countries (Table 2). Of these, 11 countries included cough, fever and weight loss or poor weight gain in their algorithms. The presence of a variety of additional symptoms and signs were also specified: fatigue, wheeze, neck mass, abdominal mass, ascites, diarrhoea, loss of appetite and night sweats. The exclusion algorithm was not defined in the remaining countries.

For people living with HIV, 86.7% (26/30) of the high-burden countries analysed provided preventive treatment for latent tuberculosis without testing for infection. In South Africa the recommendation was for a tuberculin skin test before starting preventive treatment, but this was not specified by the remaining countries. The majority of the countries (20/30) applied the WHO four-symptom screening rule (current cough, fever, weight loss and night sweats) for excluding pulmonary tuberculosis before starting preventive treatment (Table 2). Five countries specified a different set of symptoms and another five countries did not specify the symptoms to be used in the exclusion algorithm.

For children older than 12 months living with HIV, 66.7% (20/30) of high-burden countries had a recommendation for symptomatic screening alone before starting preventive treatment. Only India had a policy of doing a tuberculin skin test in addition to symptomatic screening before starting such treatment. In Angola, the recommendations were for chest radiography in addition to symptomatic screening. Only eight (26.7%) countries followed the WHO recommendation to exclude active tuberculosis based on poor weight gain, fever, current cough or contact history with a tuberculosis case (Table 2).

#### Treatment recommendations

WHO recommends 6 months of isoniazid monotherapy both for people living with HIV and children with a household contact in high-burden countries (Table 1). Among the high-burden countries reviewed, the majority (18/30) of guidelines recommended 6 months of isoniazid monotherapy, while in six countries (Cambodia, Democratic Republic of Congo, Namibia, Thailand, Viet Nam and Zimbabwe) it was a course of 6‒9 months. Central African Republic had a policy of 3 months of rifampicin plus isoniazid, as well as 6 months of isoniazid (Table 4). In Uganda and Pakistan recommendations were for an additional course of prolonged isoniazid treatment (12 and 36 months, respectively) for people living with HIV who have tuberculosis contact history. In South Africa the recommendations were 6‒36 months of isoniazid treatment, depending on the results and availability of tuberculin skin testing. In Malawi the policy was continuation of isoniazid treatment for those not receiving antiretroviral therapy but discontinuation once therapy is started.

#### Monitoring and evaluation indicators

Of the high-burden countries, only Kenya, Malawi, South Africa and Thailand had guidelines that defined indicators to evaluate the coverage of tuberculosis screening and preventive treatment among children younger than 5 years with a household contact. Most countries (18/30) defined an indicator for coverage of preventive treatment in people living with HIV (Table 4). In 2017, 10 of these countries reported data to the *Global tuberculosis report*^1^ about the proportion of patients newly enrolled in HIV care who were provided with tuberculosis preventive treatment (Table 5). Fifteen countries included information on recording and reporting tools for isoniazid preventive treatment in their guidelines (Table 4).

**Table 5:** Tuberculosis preventive treatment for people newly enrolled in human immunodeficiency virus (HIV) care in countries with the highest burdens of tuberculosis or HIV-associated tuberculosis, 2016

Country	No. (%) of people living with HIV who were newly enrolled in HIV care^a^			Indicator defined in national policy^b^
	Total	Provided with tuberculosis preventive treatment	Diagnosed with active tuberculosis	
Cambodia	3 193	631 (19.8)	N/A	Yes
Ethiopia	36 761	19 244 (52.3)	2 165 (5.9)	Yes
India	174 125	8 135 (4.7)	21 032 (12.1)	No
Indonesia	36 294	877 (2.4)	9 792 (27.0)	Yes
Liberia	4 528	390 (8.6)	1 219 (26.9)	N/A
Malawi	145 117	72 446 (49.9)	2 402 (1.7)	Yes
Mozambique	315 712	162 646 (51.5)	N/A	Yes
Myanmar	34 765	1 018 (2.9)	3 960 (11.4)	Yes
Nigeria	216 293	62 781 (29.0)	14 794 (6.8)	Yes
Philippines	5 966	2 938 (49.2)	1 645 (27.6)	No
Sierra Leone	17 843	3 609 (20.2)	1 627 (9.1)	No
South Africa	751 620	385 932 (51.3)	N/A	Yes
Swaziland	138 016	21 320 (15.4)	2 342 (1.7)	Yes
United Republic of Tanzania	49 351	4 202 (8.5)	N/A	Yes
Viet Nam	13 593	3 474 (25.6)	N/A	No
Zimbabwe	168 968	123 846 (73.3)	9 176 (5.4)	No

N/A: data not available.

^a^ Data are from the World Health Organization *Global tuberculosis report*.^1^

^b^ Indicator for coverage of preventive treatment in people living with HIV was defined in the national policy.

Note: The table includes only the 16 countries that reported data on preventive treatment among people living with HIV in 2017.

### Low-burden countries

#### Risk groups defined 

**Fig. 2:**
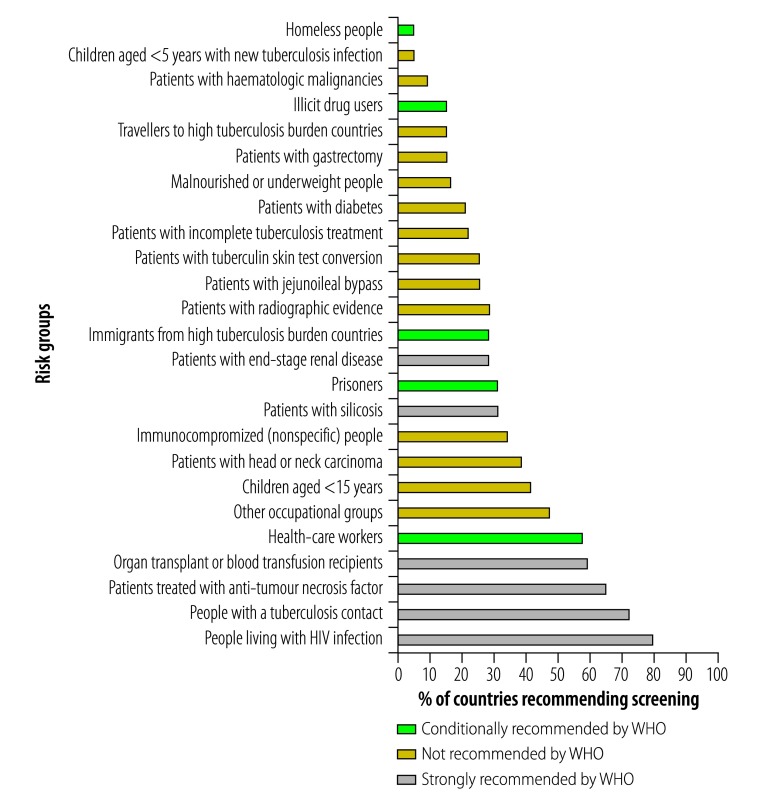
Compliance of national policies with World Health Organization guidelines on screening for latent tuberculosis infection among high-risk population groups in low-burden countries

The risk groups strongly recommended by WHO to be targeted for latent tuberculosis infection screening (Table 1) were included in the national latent tuberculosis infection policies of between 19 (27.9%) and 54 (79.4%) of 68 low-burden countries (Fig. 2). Specifically, 28 countries (42.1%) had a recommendation to screen children younger than 5 years who are contacts of a tuberculosis case. An additional 49 countries (70.1%) had recommendations to screen all contacts of a tuberculosis case, making no distinction between adults and children. For people living with HIV, the policy in 54 (79.4%) countries was to screen people living with HIV for latent tuberculosis infection and in 23 (33.8%) countries it was to screen immunocompromised individuals, which includes people living with HIV.

WHO: World Health Organization.

Notes: WHO *Guidelines on screening for latent tuberculosis infection*^11^ are summarized in Table 1. Risk groups targeted by fewer than 3% of low-burden countries are not shown on the chart. The risk categories not shown are: people with harmful alcohol use; patients with incomplete tuberculosis treatment; elderly people; children born to tuberculosis-positive mothers; and tobacco smokers. The total number of countries analysed was 68.

In contrast, some of the conditionally recommended categories (such as prisoners and illicit drug users; Table 1) were rarely mentioned in policies (Fig. 2). Notably, some countries included categories that are not recommended by the WHO; nine countries (13.2%) recommended tuberculosis preventive treatment for travellers to high tuberculosis burden countries and 10 (14.7%) for patients undergoing abdominal surgery. 

#### Testing recommendations

**Fig. 3:**
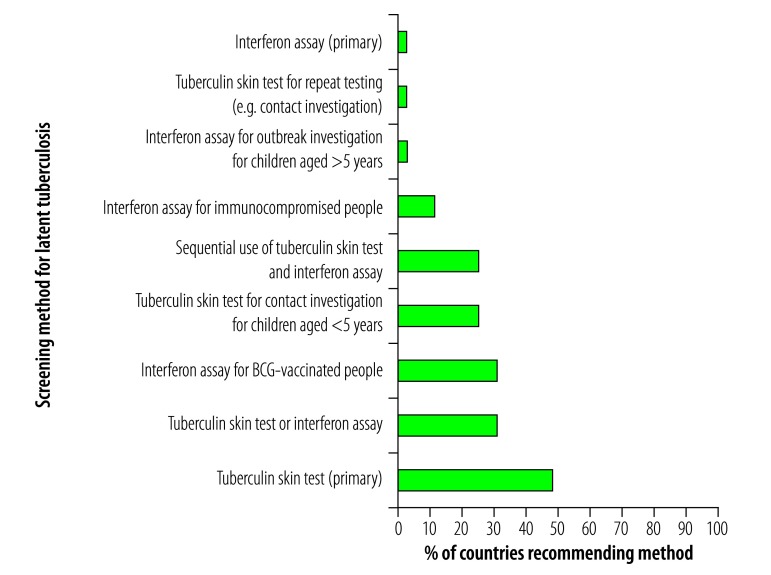
Recommendations for screening methods for latent tuberculosis infection in the national policies of low-burden countries

BCG: bacille Calmette-Guérin; WHO: World Health Organization.

Notes: World Health Organization guidelines are that either tuberculin skin test or interferon-gamma release assay can be used to screen for latent tuberculosis infection.^11^ Interferon assay refers to interferon-gamma release assay. The total number of countries analysed was 68.

The WHO latent tuberculosis infection guidelines indicate that in low-burden countries either a tuberculin skin test or interferon-gamma release assay can be used for diagnosis (Table 1). Of the low-burden countries 33/68 (48.5%) had a recommendation to use tuberculin skin testing as the primary screening method compared with only 2/68 (2.8%) recommending interferon assay (Fig. 3). In 21 countries (30.8%), the policy was either tuberculin skin test or interferon assay as the primary method of screening. In addition, multiple policies specified situations when using one test over the other was preferable. For example, in 21 (30.8%) countries the policy was that interferon assay should be used for individuals vaccinated with bacille Calmette–Guérin (BCG) and in 17 (25.0%) countries that interferon assay and tuberculin skin test should be used sequentially. For some countries, including Costa Rica and Uruguay, there were no explicit recommendations on methods of testing.

An algorithm for excluding active tuberculosis was specified in the policies of 43 (63.2%) low-burden countries, although the content of that algorithm varied greatly from country to country. In Colombia, Ecuador and Uruguay the recommendation was only that active tuberculosis should be ruled out, with no mention of an exclusion algorithm. All other countries required at least a chest X-ray.

#### Treatment recommendations

**Fig. 4:**
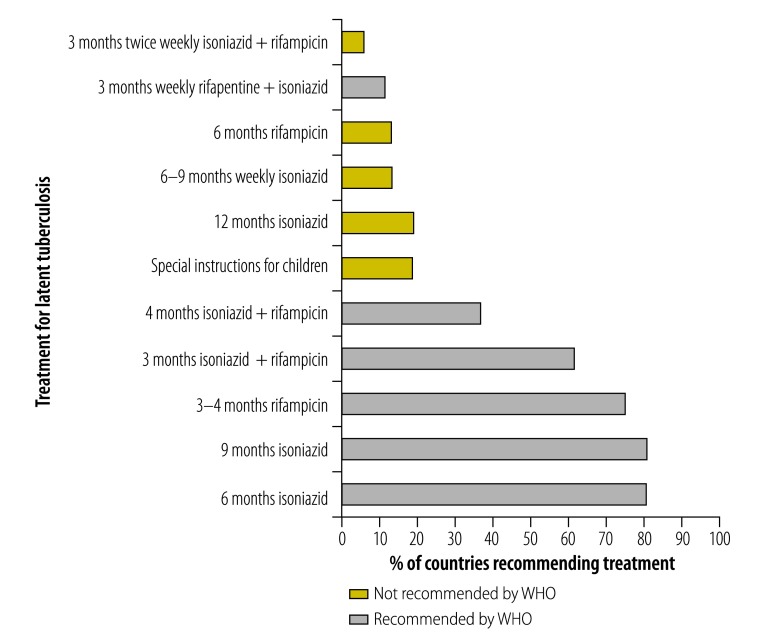
Compliance of national policies with World Health Organization guidelines on treatment of latent tuberculosis infection in low-burden countries

The most commonly recommended treatments in low-burden countries were isoniazid for 6 months (55 countries; 80.8%) or 9 months (55 countries, 80.8%) (Fig. 4), which is in line with the WHO guidelines on treatment of latent tuberculosis (Table 1). Alternative treatment options recommended by the WHO were also frequently mentioned in other policies, but to a lesser extent, ranging from 8 (11.7%) to 51 (75.0%) countries.

WHO: World Health Organization.

Notes: WHO guidelines on treatment of latent tuberculosis infection^11^ are summarized in Table 1. Special instructions for children refer to any policy recommendation for an alternative treatment or duration of treatment for children younger than 5 years. The total number of countries analysed was 68.

#### Monitoring and evaluation indicators

Monitoring and evaluation of latent tuberculosis infection screening was mentioned in the policies of 32 (47.1%) low-burden countries. Even among the countries that mentioned reporting requirements, those were often specific to active tuberculosis, and therefore the form may be inappropriate for latent tuberculosis infection.

## Discussion

This review identified that the majority of both high- and low-burden countries had a national policy that addressed latent tuberculosis infection management in people living with HIV and children younger than 5 years with a household contact. Clinical high-risk groups were also covered by most guidelines from low-burden countries. However, the content of the guidelines varied considerable across countries. For example, clear and standard algorithms for excluding tuberculosis before treatment and latent tuberculosis infection testing were not available in many countries, and indicators for monitoring and evaluation were rarely defined. Guidelines are the first step in implementing the programmatic management of latent tuberculosis infection, hence it is essential to provide clear and simple operational guidance, including evidence-based standardized algorithms and a framework of monitoring and evaluation.^14^

The advantage of an evidence-based standardized algorithm was demonstrated by the WHO recommended four-symptom screening rule to exclude active tuberculosis before starting preventive treatment for people living with HIV.^10^ This simple algorithm adds to the clarity of the policy and has resulted in a steep rise in implementation of isoniazid preventive treatment among people living with HIV in settings with a high prevalence of tuberculosis and low resources, reaching 1.3 million in 2016.^1^ Ensuring that guidelines and algorithms are simple can also facilitate their incorporation into national guidelines. For example, seven out of 10 countries that had algorithms different from the WHO recommendation in a previous policy review^15^ have now adopted them (Cameroon, Lesotho, Nigeria, South Africa, Swaziland, United Republic of Tanzania and Viet Nam).

In contrast to the uptake of the screening algorithm for people living with HIV, the corresponding screening algorithm for children was not taken up or defined in national policies. This could be due to the limited evidence about the effectiveness of the algorithm, as it was recommended largely based on expert opinion.^16^ Further research is needed to evaluate the performance of the algorithm and identify the optimal approach to exclude active tuberculosis in children before starting preventive treatment.

Consistent with our previous study,^13^ we found that the national policies and guidelines in the majority of low-burden countries addressed latent tuberculosis infection specifically or as part of the general tuberculosis policy. The Netherlands has revised its guidelines since the publication of the 2015 WHO latent tuberculosis infection guidelines,^17^ which are now mostly consistent with WHO recommendations. A similar revision by other countries would increase alignment between national policies and WHO recommendations. This could lead to more consistent and comprehensive latent tuberculosis infection policies and pave the way for global monitoring and evaluation of the programmatic management of latent tuberculosis infection. Although it may be too early to evaluate the impact of such policy changes on tuberculosis incidence, it is a question that needs to be addressed in the future.

Tuberculin skin testing was the most frequently recommended diagnostic tool. The test requires no laboratory work and is comparably cheaper per unit test than interferon-gamma release assay. That may explain the overwhelming preference for the test over interferon assay in the policies of low-burden countries. Several countries specified additional diagnostic algorithms, such as different tuberculin skin test cut-off points among specific risk groups, sequential use of the two tests, or use of interferon assay for BCG-vaccinated individuals. A systematic review did not show a significant difference in the prediction of progression to active tuberculosis between the two tests in head-to-head analysis.^11^ However, there were insufficient data on the predictive utility among specific populations. The diversity of policies across countries calls for more research in how to use interferon-gamma release assay and tuberculin skin testing together among different risk groups based on the underlying tuberculosis epidemiology.

This policy review has limitations. First, determining the latest published guidelines was done through contacting national programmes, WHO offices and through extensive internet searches; however some policies may not have been identified. Even though latent tuberculosis infection monitoring and evaluation indicators may not have been defined in guidelines they may nevertheless exist within a country’s national tuberculosis programme or other guidelines. These limitations might have led to misclassification of the findings. Second, a single person was responsible for reviewing policies, extracting relevant information and entering data within each group (high-and low-burden countries). While this provided internal consistency, the data collection may have been subject to reviewer bias.

In conclusion, our review identified large variations across countries in their national tuberculosis policies. The differences are probably attributable to different country contexts and disease epidemiology and lack of consensus on some aspects of latent tuberculosis infection management. There are unique challenges associated with management of latent tuberculosis infection, such as exclusion of active tuberculosis, testing for latent tuberculosis infection and treatment initiation. It is therefore important to continue to develop clear, implementable and evidence-based WHO policies. An important component of such policies should be monitoring and evaluation, as this is essential to assess progress in the implementation and to make policy decisions. Lack of a monitoring and evaluation component in more than half of the national policies presents a barrier to programmatic management of latent tuberculosis infection.
